# Activation of the Insulin Receptor by *Sarcopoterium spinosum* Extract and Identification of Sarcocyanidin A as a Novel Active Compound

**DOI:** 10.1021/acsomega.5c00451

**Published:** 2025-04-09

**Authors:** Ayala Wollman, Rania Hasib Afana, Shmuel Carmeli, Tovit Rosenzweig

**Affiliations:** a Department of Molecular Biology, Faculty of Natural Sciences, Ariel University, Ariel 40700, Israel; b Raymond and Beverly Sackler School of Chemistry, Faculty of Exact Sciences, Tel Aviv University, Ramat Aviv, Tel Aviv 69978, Israel; c The Adelson School of Medicine, Ariel University, Ariel 40700, Israel

## Abstract

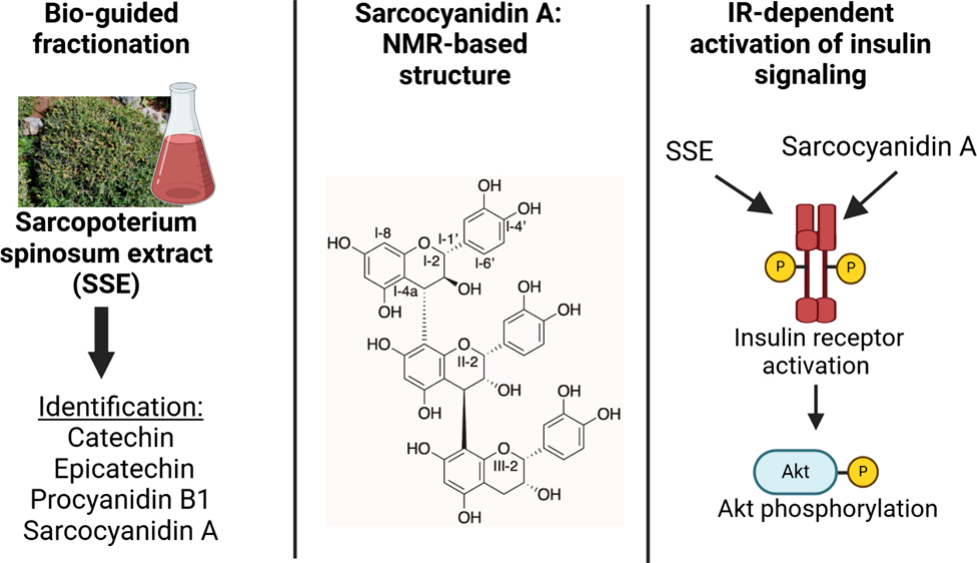

*Sarcopoterium spinosum* is a medicinal plant, presenting glucose-lowering properties. The study aimed to identify the active components and their mechanisms of action. Bioguided fractionation was utilized to isolate the active molecules, followed by NMR and HRESI MS for their identification and structural elucidation. Binding to the insulin receptor (IR) and activation of the receptor were measured *in vitro*. Glucose-lowering effects were validated *in vivo*. A novel procyanidin trimer, named sarcocyanidin A (**1**, catechin-(4α-8)-epicatechin-(4β-8)-epicatechin), was identified. Sarcocyanidin A (**1**) activated insulin signaling in CHO-IR and L6 myotubes, while the IR inhibitor abolished this effect. IR autofluorescence and cell-based thermal shift assays indicate a direct interaction of sarcocyanidin A (**1**) with IR. Sarcocyanidin A (**1**) also activated insulin signaling and reduced blood glucose in mice. Sarcocyanidin A, a novel procyanidin trimer, mediates at least part of the antidiabetic properties of SSE, through activation of IR.

## Introduction

Type 2 diabetes (T2D) is a prevalent condition affecting a substantial global population, as indicated by recent epidemiological data.^1,2^ The disease is characterized by insulin resistance and glucose intolerance, which lead to chronic hyperglycemia. When left unmanaged, T2D might lead to severe complications and stands as a major risk factor for the development of various morbidities, such as cardiovascular and fatty liver diseases.^3,4^ Despite the growing array of therapeutic options, marked by the introduction of new antidiabetic drugs, a considerable proportion of patients still do not achieve the therapeutic target for HbA1C levels.^5^ Importantly, there is currently no medication designed to target insulin resistance, which is a major pivotal pathology contributing to the progression of T2D.

*Sarcopoterium spinosum* (S. spinosum) is a plant utilized by traditional Bedouin medicinal practitioners for the treatment of diabetes.^6,7^ Several compounds, such as dicatechins, tormentic acid, hydroxytormentic acid, and ursolic acid, have been identified in its extract.^8,9^ However, the specific functions of these compounds and their contribution to the antidiabetic properties of the extract remain poorly explored.^10^

In our previous studies, we were able to show that *Sarcopoterium spinosum* root extract (SSE) can alleviate elevated blood glucose levels in mouse models of glucose intolerance.^11−13^ This was achieved either by enhancing insulin sensitivity or by mimicking the effect of the hormone.^14,15^ Our previous studies also demonstrated that SSE stimulated the transmission of the insulin signaling cascade in L6 and 3T3-L1 adipocytes. In addition, it was shown that SSE augmented glucose uptake in adipocytes through a PI3K-AKT-dependent mechanism.^15^ Nevertheless, the specific component of insulin signaling, which is targeted by SSE, has not yet been identified yet. Accordingly, the present study is designed to identify the active components present in SSE and to illuminate their mechanisms for activating insulin signaling. Specifically, the effect of SSE on events occurring upstream of PI3K-AKT, mainly phosphorylation of IR, was investigated. To identify the active components of SSE and to assess the role of IR in mediating SSE’s effects on the insulin signaling cascade, we employed IR-overexpressing CHO cells, which are highly sensitive for activators of insulin receptors, in addition to L6 and 3T3-L1 adipocytes. The active components of SSE were isolated through biological activity-guided fractionation of the extract.

## Experimental Section

### Materials

IBMX, dexamethasone, insulin, 2-deoxy-d-glucose (2-DG), cytochalasin-B, gallic acid, folin, and Ciocalteu’s phenol reagent and inhibitors of proteases and phosphatases were all purchased from Merck. BSA and the media for cell cultures were obtained from Biological Industries (Beit Haemek, Israel). [^3^*H*]-2-Deoxy-d-glucose (1 mCi) and OptiPhase scintillation solution were purchased from PerkinElmer. Hygromycin B was purchased from Thermo Fisher Scientific. A recombinant insulin receptor was ordered from R&D Biosystems. HNMPA-(AM)3 was ordered from Abcam. Tormentic acid, ursolic acid, and procyanidins (PCs) were purchased from Cayman. Antiactin was obtained from MP Biomedicals. Other primary antibodies were obtained from Cell Signaling Technology. Secondary antibodies were purchased from Jackson ImmunoResearch.

### Methods

#### SSE Preparation

*S. spinosum* root extract was prepared from the plant (*Sarcopoterium spinosum* (L.) sp. Rosaceae family) roots, collected from the wild area around Ariel, Israel. The plants were identified by the botanical staff of the University (a voucher specimen was deposited in the Israel National Herbarium at the Hebrew University of Jerusalem, HUJ 102531). The SSE was prepared and lyophilized as described before.^11^ The dose for *in vitro* and *in vivo* studies was selected according to previous publications^11,15^ and cytotoxicity measurements.^16^

#### Chemical Characterization: Mass Spectrometry

SSEs of samples collected from the wild area nearby Ariel, Israel, over several months were dissolved in dideionized water (DDW), filtered through a 0.22 μm PTFE filter, and injected to the LCMS. The HRESILCMS spectra were recorded on a Waters (Milford, MA, USA) Xevo G2-XS QTOP instrument equipped with an Acquity Hi Class UPLC (binary solvent manager) with an FTN sample manager, a column manager, and a PDA UV detector, using a 2.1 × 50 mm BEH C18 (1.7 μm) column and a flow of 0.3 mL/min. Samples of 10 μL were injected and eluted with a gradient of solvents composed of 100% H_2_O + 0.1% FA (solvent A) and acetonitrile + 0.1% formic acid (solvent B). The elution started with 100% solvent A for 5 min then a linear increase to 100% solvent B over 20 min and then returned to the starting conditions for an additional 2 min.

#### Bioactivity-Guided Isolation of Sarcocyanidin A (**1**), Epicatechin (**2**), Catechin (**3**), and Procyanidin B1 (**4**)

**Figure 1:**
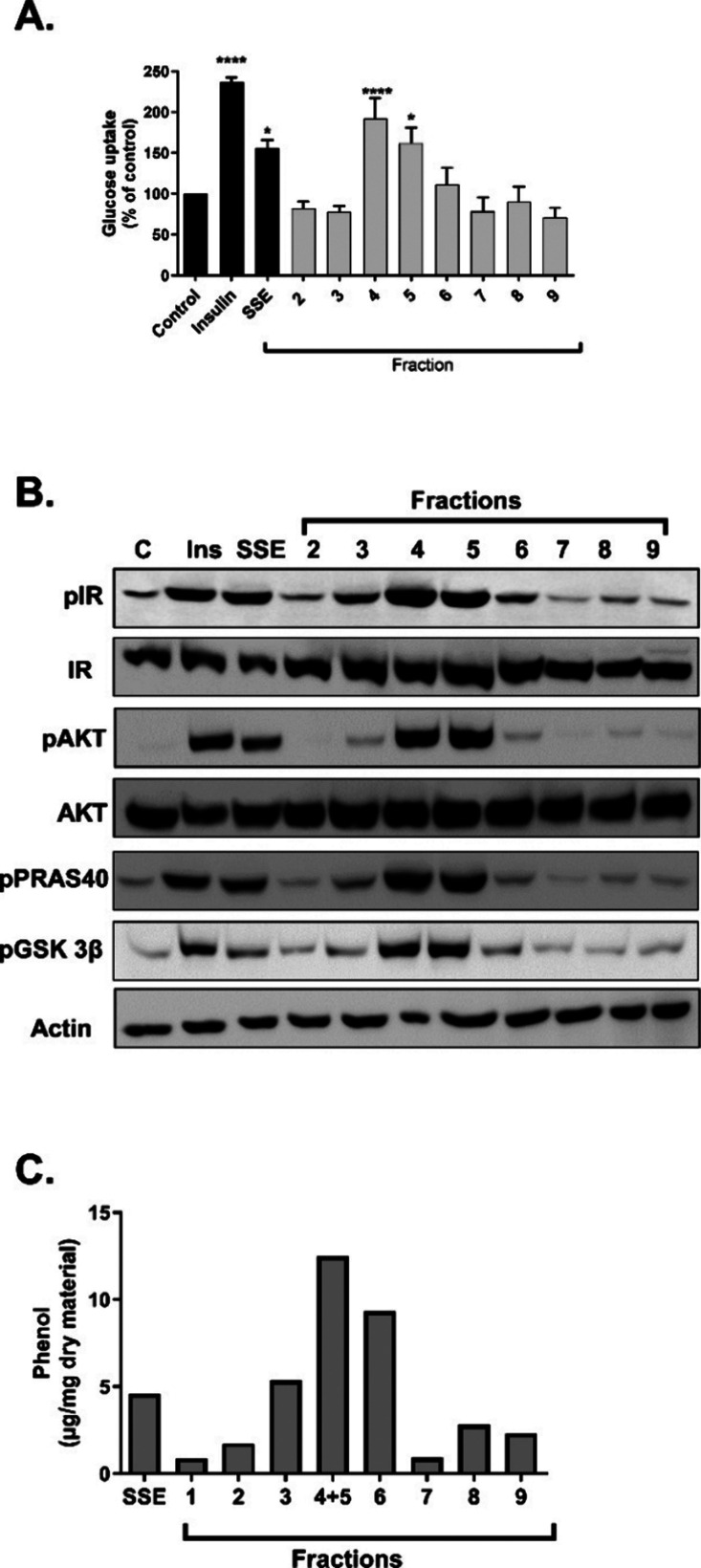
Active components of SSE are found within the phenol-rich fractions. SSE was separated based on polarity on a C18 column to nine fractions. The activity of the fractions was measured by three different bioassays. (A) Differentiated 3T3-L1 adipocytes were treated with SSE or its fractions at 100 μg/mL, using insulin (100 nM) as a positive control. [^3^*H*]-2-Deoxy-d-glucose uptake into cells was determined as described in the Materials and Methods. (B) L6 myotubes were treated with 100 nM insulin, SSE, or its fraction (100 μg/mL) for 10 min. Western blot analysis of the whole lysate was performed using specific antibodies. (C) Phenolic content in the SSE fractions was determined by the Folin-Ciocalteu method. The data represent the mean ± SEM of measurement made on at least three independent experiments. *p < 0.05, ****p < 0.0001 compared to untreated cells by one-way ANOVA, followed by Tukey’s post-testing.

SSE (22.6 g) was separated on a CombiFlashEZ Prep equipped with a reversed-phase C18 column (Teledyne ISCO, HP C18Aq, 150 g) in 10 consecutive portions (Scheme S1). The SSE (2.26 g) was dissolved in 150 mL of dideionized water (DDW), absorbed on HP C18 (20 g), evaporated to dryness, and packed in a precolumn. The precolumn was installed on top of the column and eluted with a linear gradient from 100% water to 100% methanol and then to 100% ethyl acetate within 60 min at a flow of 20 mL/min. The eluent was collected by a sample autocollector to 45 tubes, which were initially combined by the UV absorption into 17 fractions and after examination of their ^1^H NMR spectra were further combined into nine fractions. Fractions 4 and 5 (Scheme S1), which contained catechin monomers and oligomers and presented a higher percentage of glucose uptake than the SSE (as presented in Figure 1), were thus further separated by size-exclusion chromatography on a Sephadex LH-20 equilibrized and eluted with 1:1 water/methanol. Fraction 4 (4.38 g) was separated in five consecutive portions on a Sephadex LH-20 (250 mL, Φ × *h*, 3.4 cm × 28 cm) to yield 15 fractions. Fractions 8 (65.1 mg) and 9 (230.5 mg) from this separation process contained an almost pure procyanidin trimer. Fraction 8 was purified on a preparative reversed-phase HPLC column (YMC Pack ODS-A, 10 μm, 250 × 20 mm) eluted under isocratic conditions (9:1 aq. formic acid/acetonitrile, 5 mL/min) to produce pure active sarcocyanidin A (**1**) (3.0 mg, *R*_t_ 16.5 min). While collecting the NMR data of **1** overnight, we noticed that **1** was converted to another product; thus, we examined other separation conditions (without formic acid) and the HPLC column for the separation of fraction 9. Finally, fraction 9 was purified on a preparative reversed-phase HPLC column (Phenomenex Luna 5 μm, phenyl-hexyl 100 A, 250 × 21.2 mm) eluted under isocratic conditions (9:1 water/acetonitrile, 5 mL/min) to produce pure active sarcocyanidin A (**1**) (10.1 mg, *R*_t_ 23.5 min). Fraction 5 from the initial separation (7.83 g) was separated in seven consecutive portions on the Sephadex LH-20 column to afford in fraction 8 (out of 14 fractions) an almost pure catechin derivative (16.2 mg) that was identified by 1D and 2D NMR and HRESI MS to be *epi*catechin (**2**). Combined fractions 6 and 7 (450 mg) from the latter Sephadex LH-20 column were separated on a preparative reversed-phase HPLC column (YMC Pack ODS-A, 10 μm, 250 × 20 mm) eluted under isocratic conditions (85:15 water/acetonitrile, 10 μm, 5 mL/min) to produce in fraction 5 (22.8 mg, *R*_t_ 11.3 min) a pure catechin monomer (**3**) and in fraction 7 (13.6 mg, *R*_t_ 13.7 min) a pure catechin dimer (**4**). Compound **3** was identified by 1D and 2D NMR and HRESI MS to be catechin, while compound **4** was identified by 1D and 2D NMR and HRESI MS to be procyanidin B1.

##### Sarcocyanidin A (**1**)

[α]_D_^20^ −38.0 (*c* 0.050, H_2_O); UV (H_2_O) λ_max_ (log ε): 279 (4.4) nm; IR (ATR diamond): ν_max_ 3233br, 2912, 1708 cm^–1^; for ^1^H and ^13^C NMR data, see Table 1; HRESIMS [M–H]^−^, *m*/*z* 865.1970 (calc for C_45_H_38_O_18_, 865.1985).

**Table 1:** Retention Times and Molecular Ions of Typical Components of the SSE

**compound**	*R*_t_	**[M–H]**^**–**^
	6.77	1027
sarcocyanidin A	7.03	865
	7.42	951
	7.65	365
	7.95	577
procyanidin B1	8.15	577
	8.26	865
catechin	8.51	289
epicatechin	9.56	289
	10.20	577
	11.58	263
	11.69	395
	12.14	265
23-HTAEG^a^	13.04	711^e^
	13.30	711
3-Gal-23-HTAEG^b^	13.46	817
23-HTAEG-24-oate^c^	14.79	695
23-HTA^d^	15.07	503

a 23-HTAEG: 23-hydroxytormentic acid ester glycoside.

b 3-Gal-23-HTAEG: 3-galoyl-23-hydroxytormentic acid ester glycoside.

c 23-HTAEG-24-oate: 23-hydroxytormentic acid ester glycoside-24-oate.

d 23-HTA: 23-hydroxytormentic acid.

e [M+formate (45)].

##### *Epi*catechin (**2**)

[α]_D_^20^ −12.4 (*c* 0.098, MeOH); UV (MeOH) λ_max_ (log ε): 280 (3.2) nm; IR (ATR diamond): ν_max_ 3231br, 2929, 1607, cm^–1^; for ^1^H and ^13^C NMR data, see Table S1 in the Supporting Information; HRESIMS [M–H]^−^, *m*/*z* 289.0718 (calc for C_15_H_13_O_6_, 289.0712).

##### Catechin (**3**)

[α]_D_^20^ 57.1 (*c* 0.029, MeOH); UV (MeOH) λ_max_ (log ε): 279 (3.2) nm; IR (ATR diamond): ν_max_ 3256br, 2919, 1610, cm^–1^; for ^1^H and ^13^C NMR data, see Table S2 in the Supporting Information; HRESIMS [M–H]^−^, *m*/*z* 289.0711 (calc for C_15_H_13_O_6_, 289.0718).

##### Procyanidin B1 (**4**)

[α]_D_^20^ 91.4 (*c* 0.013, H_2_O); UV (H_2_O) λ_max_ (log ε): 279 (4.1) nm; IR (ATR diamond): ν_max_ 3377br, 2922, 1610, cm^–1^; for ^1^H and ^13^C NMR data, see Table S3 in the Supporting Information; HRESIMS [M–H]^−^, *m*/*z* 577.1351 (calc for C_30_H_25_O_12_, 577.1346).

### Total Polyphenol Content Analysis

Polyphenol levels were measured using the Folin-Ciocalteu method.^17^ The fraction (25 μL) was added to the same volume of a Folin-Ciocalteu reagent and 200 μL water. The mixture was allowed to equilibrate for 5 min and then mixed with 100 μL of sodium carbonate solution (20%). The mixture was incubated at 37 °C for 40 min, and the absorbance was read at 750 nm. A standard curve was prepared with gallic acid (5–40 mg/mL). The results were expressed as μg of catechin equivalents per milligram of dry material.

### Cell Culture

3T3-L1 preadipocytes (ATCC) were cultured and induced to differentiate as described before.^14^ L6 myoblasts (ATCC) were grown in MEM-α containing 25 mM glucose, 10% FCS, 2 mM glutamine, and 1% ampicillin. Experiments were performed on differentiated myotubes. L6 differentiation was induced, as described in our previous study.^14^ CHO cells overexpressing the insulin receptor (CHO-IR) were ordered from ATCC (ATCC CRL-3307). Cells were maintained in growing media (F-12K medium containing 1.26 g/L d-glucose, glutamine (2 mM), pen-strep (0.9%), inactivated fetal bovine serum (10%), and hygromycin B (0.3 mg/mL). For experiments, cells were seeded at a concentration of 2.5 × 10^5^ cell/mL, in media without hygromycin B. Cells were starved for 18 h before analysis in media lacking serum and hygromycin B.

### Glucose Uptake

Differentiated adipocytes were preincubated for 2 h in serum-free DMEM. Starvation media was replaced, and cells were treated with SSE or its fractions (100 μg/mL) for 30 min. Insulin (100 nM) was used as the positive control. Glucose uptake was measured as described in our previous publication.^15^

### Preparation of Cell Lysates and Western Blot Analysis

Protein lysates of CHO-IR cells, L6, and 3T3-L1 cells were prepared using RIPA buffer supplemented with protease and phosphatase inhibitors (Merck) as described (ref). The Bradford method was utilized to measure protein concentration. Protein was separated (20 μg per lane) by SDS-polyacrylamide gel electrophoresis as described before.^15^

### Measurement of pIR by ELISA

L6 myotubes were treated with insulin (100 nM) or SSE (100 μg/mL) for 10 min. The protein lysate was prepared, and tyrosine phosphorylation of IR was measured by an ELISA kit (phospho-insulin receptor β sandwich ELISA, C7082, Cell Signaling Technologies) according to the manufacturer’s instructions.

### Protein Tyrosine Phosphatase Activity Assay

L6 myotubes were treated with insulin (100 nM) or SSE (100 μg/mL) for 5 and 10 min; cells were washed twice in cold PBS, scraped in the presence of antiproteases and DTT (2 mM), incubated in ice for 5 min, and vortexed. Cells were centrifuged (4 °C, 10,000*g*, 15 min), and the supernatant was collected. Phosphatase activity was measured with a protein phosphatase assay kit (Abcam, ab241032), according to the manufacturer’s instructions. Suramin and phosphatase inhibitors were used as the positive control and negative control, respectively.

### Cellular Thermal Shift Assay

The assay was done based on the protocol described by Jafari et al.^18^ CHO-INSR cells were treated for 10 min with insulin, SSE, or trimer catechin. Cells were washed with cold PBS, scraped gently, and centrifuged (300*g*, 3 min). Cells were then washed in PBS and centrifuged again. Cells were then diluted in 1 mL of PBS, containing antiproteases. The cell suspension was divided into 10 Eppendorf tubes, and each was incubated in a PCR machine for 3 min at different temperatures (43–52 °C). Cells were immediately transferred to ice and lysed by 2 cycles of freeze-and-thaw. Cells were centrifuged (4 °C, 20,000*g*, 20 min), and sample buffer was added to the supernatant. The samples were incubated at 70 °C for 10 min, and gel electrophoresis was performed for the detection of the IR expression level.

### Fluorescence Quenching Measurement

This measurement is based on autofluorescence of aromatic amino acids, which is affected by the protein structure and its binding to small molecules. Fluorescence of IR (0.3 μM) in phosphate buffer (pH 7.4) was measured (Ex/Em = 280/300–500 nm) in the presence or absence of SSE or sarcocyanidin A (**1**). Fluorescence of denaturized IR was measured by the addition of guanidine (1 M) to the mixture.

### Effect of the SSE, Active Fraction (Fr 4 + 5), and Trimeric Catechin on the Glucose Level in Mice

The study was carried out following the recommendations in the Guide for the Care and Use of Laboratory Animals of the National Institutes of Health and complied with the Animal Research: Reporting of In Vitro Experiments (ARRIVE) guidelines. The protocol was approved by the Committee on the Ethics of Animal Experiments of Ariel University (permit number IL-2207-124). The effect of a single dose of SSE, its active fraction (Fr 4 + 5), and trimeric catechin was measured in male C57bl/6 mice (Envigo, Israel). Experiments were conducted on male mice, which are more prone to developing obesity-related glucose intolerance than female. The mice were housed in an animal laboratory with a controlled environment of 20–24 °C, 45–65% humidity, and a 12 h light/dark cycle. SSE, Fr 4 + 5, and trimeric catechin were given (intraperitoneal) to nonfasting STD-fed mice (STD, 18% of total calories derived from fat, 24% from proteins, and 58% from carbohydrates; Harlan, Teklad TD.2018). SSE was also given to mice after 8 weeks of HFD feeding (HFD, 60% of total calories derived from fatty acids, 36% saturated, 41% monounsaturated, and 23% polyunsaturated; 18.4% from proteins; 21.3% from carbohydrates, Teklad TD.06414). *N* = 6 mice in each group. Blood glucose was measured each hour for 6 h and after 24 h. An additional experiment was performed in which mice were killed 6 h after SSE, Fr 4 + 5, or trimeric catechin administration. Liver and adipose tissues were removed, the protein lysate was prepared, and Western blot analysis for the detection of insulin signaling was conducted.

### Data and Statistical Analysis

The data and statistical analysis comply with the recommendations for experimental design and analysis in pharmacology. Values are presented as means ± SEM. Statistical differences between the treatments and controls were tested by unpaired two-tailed Student’s *t*-test or one-way analysis of variance (ANOVA), followed by Bonferroni’s post hoc testing when appropriate. Analysis was performed using GraphPad Prism 10.0 software. A difference of *p* < 0.05 or less in the mean values was considered statistically significant.

## Results

**Figure 2:**
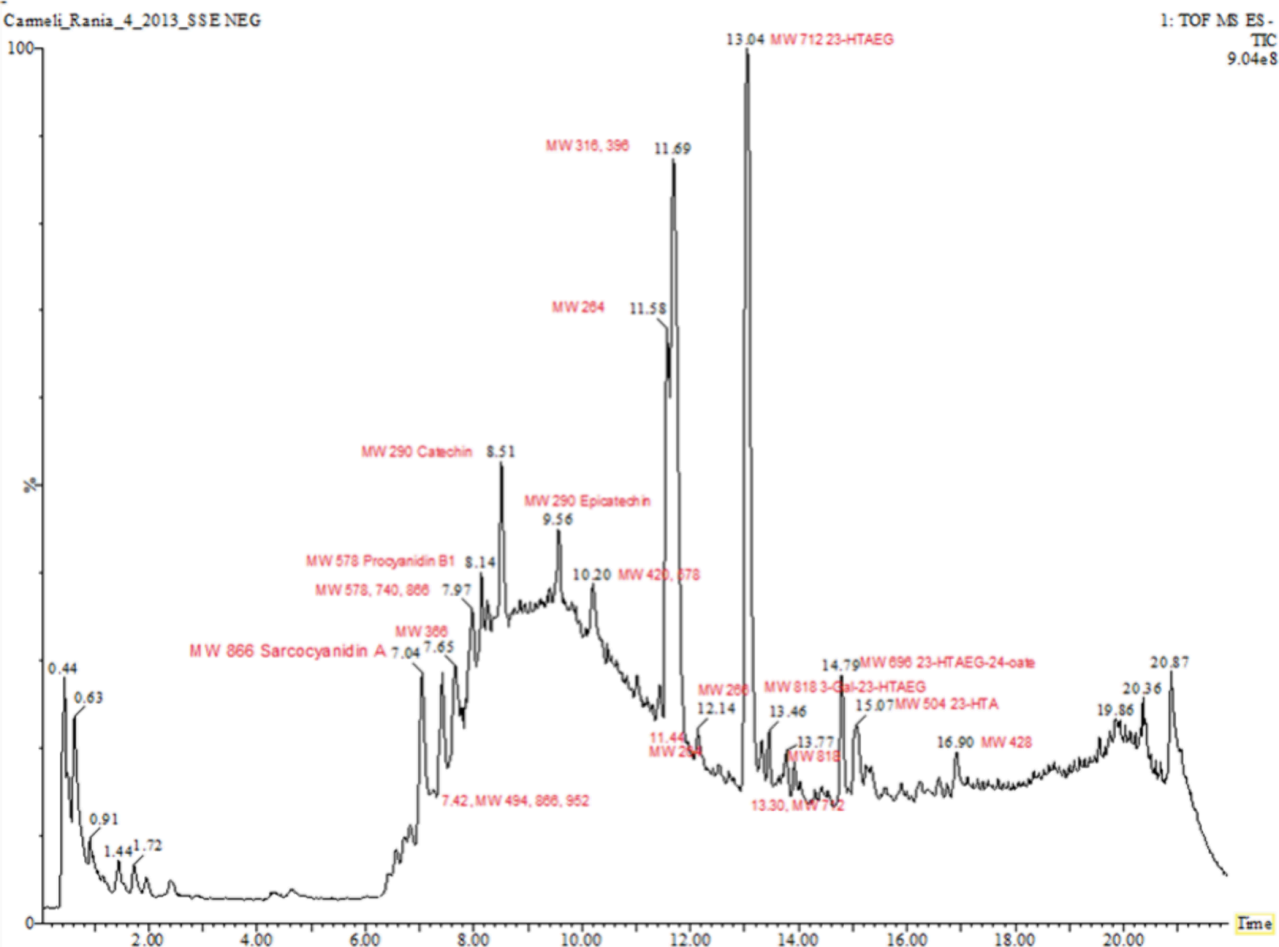
Typical ESI(−) LCMS annotated chromatogram of SSE.

Isolation and identification of active compounds were performed using the bioguided fractionation strategy (Scheme S1). The whole extract was loaded on a C18 column and fractionated by a gradient of H_2_O/MeOH. According to previous studies, the activity of the isolated fractions was characterized based on their potency in the induction of glucose transport and activation of insulin signaling.^15,16^ As presented in Figure 1, fractions 4 and 5 stimulated glucose uptake in 3T3-L1 adipocytes (Figure 1A) and activated the cascade of insulin signaling in L6 myotubes (Figure 1B), while other fractions were utterly inactive. These two active fractions were found to be rich in phenolic compounds (Figure 1C). Among these phenolics, catechin, epicatechin, and procyanidin (PC) dimers and trimers were identified by LCMS (Table 1 and Figure 2). Specifically, the active dimer was identified as the known procyanidin B1, which is the (−)-epicatechin-(4β-8)-(+)-catechin dimer, and the trimer structure was decoded to be a new trimer, (+)-catechin-4α-8-(−)-epicatechin-4β-8-(−)-epicatechin (sarcocyanidin A), as detailed below.

### Structure Elucidation of Sarcocyanidin A (**1**)

**Figure 3:**
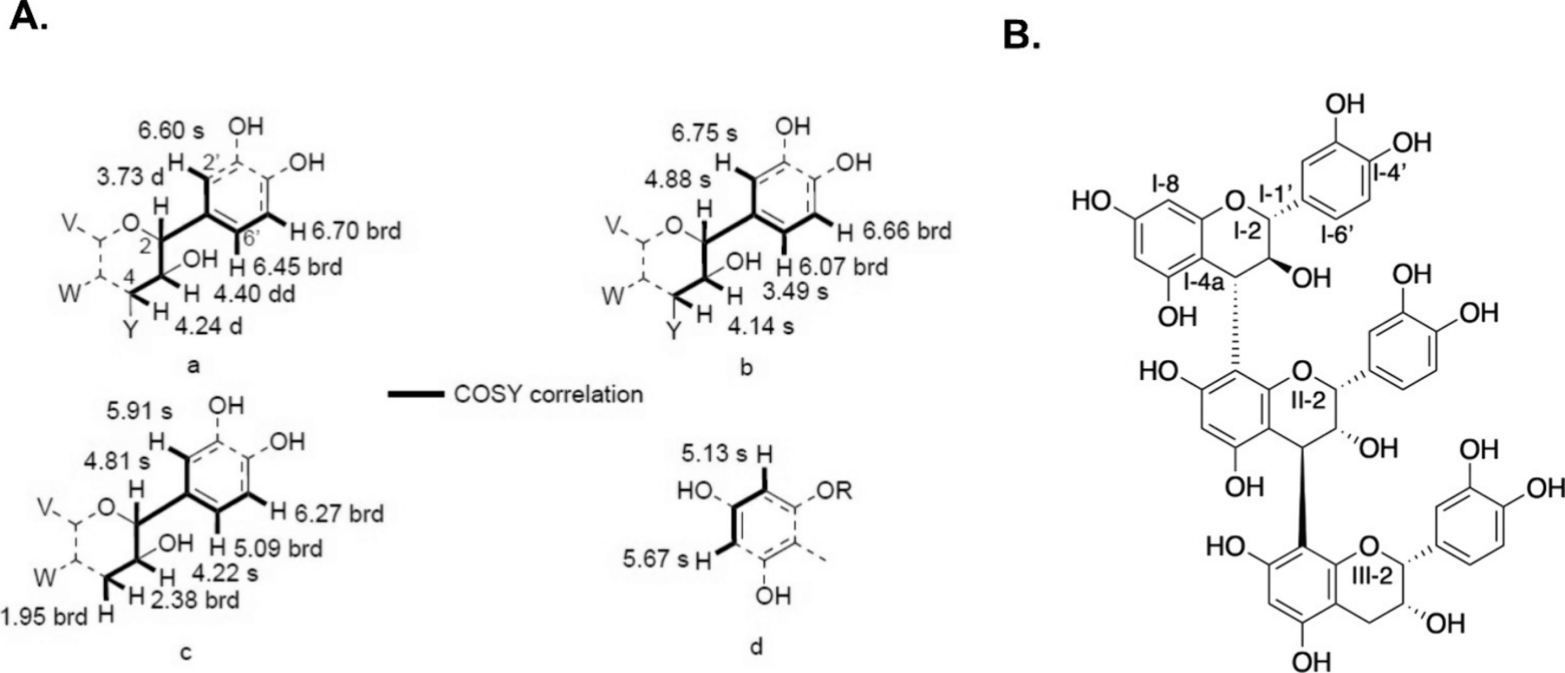
(A) Structure of fragments a–d deduced from the ^1^H–^1^H COSY correlations. (B) Structure of sarcocyanidin A (**1**).

Sarcocyanidin A (**1**) was isolated as a reddish amorphous solid, which exhibited a negative HRESIMS molecular ion, [M–H]^−^ at *m*/*z* 865.1970 corresponding to the molecular formula C_45_H_38_O_18_ and 27 degrees of unsaturation. The latter molecular formula was in agreement with that of a catechin trimer. The ^13^C NMR spectrum of **1** in D_2_O (Table 2) presented signals of 45 carbons: 18 sp^2^ quaternary carbon between 159 and 132 ppm; nine sp^2^ methine carbons between 123 and 113 ppm, five sp^2^ quaternary carbon between 111 and 101 ppm; four sp^2^ methine carbons between 99 and 96 ppm; eight sp^3^ methine carbons between 85 and 38 ppm and a single methylene carbon at 23.7 ppm. The ^1^H NMR spectrum of **1** in D_2_O (Table 2) presented signals of 13 sp^2^ methine protons between 6.70 and 5.09 ppm, six of which are broad doublets and the rest singlets, eight sp^3^ methine protons between 4.90 and 3.45 ppm, and two broad doublet protons of a single sp^3^ methylene resonating at 2.38 and 1.95 ppm. The correlation from the 2D NMR ^1^H–^1^H COSY spectrum allowed to propose four fragments (Figure 3A): (a, H-I-4 to H-I-3, H-I-3 to H-I-2, H-I-2 (^4^*J*) to H-I-2′, H-I-2′ (^4^*J*) to H-I-6′, and H-I-6′ to H-I-5′), (b, H-II-4 to H-II-3, H-II-3 to H-II-2, H-II-2 (^4^*J*) to H-II-2′ and H-II-6′, H-II-2′ (^4^*J*) to H-II-6′, and H-II-6′ to H-II-5′), (c, H-III-4 to H-III-3, H-III-3 to H-III-2, H-III-2 (^4^*J*) to H-III-2′ and H-III-6′, H-III-2′ (^4^*J*) to H-III-6′, and H-III-6′ to H-III-5′), and (d, H-I-6 (^4^*J*) to H-I-8). Two singlet sp^2^ protons did not show any correlation in the COSY spectrum. Analysis of the correlations from the HSQC and HMBC 2D spectra (Table 2 and Figure S2) allowed us to construct from the latter four fragments and the two unpaired protons three catechin-type fragments. Comparison of the *J* values of the protons connected to the sp^3^ carbons of the latter three fragments of **1** with those of epicatechin (**2**), catechin (**3**), and procyanidin C4^19^ allowed to identify the three fragments as 4α-substituted (+)-catechin, 4β,8-disubstituted (−)-epicatechin and 8-substituted (−)-epicatechin. The HMBC correlation of H-I-3 and H-I-4 with C-II-8 and of H-I-4 with C-II-8a allowed to connect the (+)-catechin to the disubstituted (−)-epicatechin and HMBC correlations of H-II-4 with C-III-7 and C-III-8 allowed to connect the disubstituted (−)-epicatechin with the (−)-epicatechin. The structure of sarcocyanidin A (**1**) was thus established as catechin-(4α-8)-epicatechin-(4β-8)-epicatechin (Figure 3B). The structure assignment was supported as well by the NOEs from a ROESY experiment (Figure S3).

**Table 2:** NMR Data of Sarcocyanidin A (**1**) in D_2_O^a^

position	δ_C_, mult.	δ_H_, mult. (*J* in Hz)	HMBC correlations^b^	COSY correlations^c^	ROESY correlations^c^
I-2	84.1, CH	3.73, d (10.0)	I-3,2′,6′	I-3,2′	I-3,4,2′,6′
I-3	74.8, CH	4.40, dd (10.0, 7.8)	I-2,3	I-2,4	I-2,2′,6′, III-2
I-4	39.0, CH	4.24, d (7.8)	I-2,3	I-3	I-2,2′,6′
I-4a	109.6, C		I-4		
I-5	158.4, C		I-4		
I-6	96.7, CH	5.67, s		I-8	
I-7^d^	156.1, C				
I-8	96.4, CH	5.13, s		I-6	
I-8a	155.2, C		I-4		
I-1′	133.1, C		I-2,3,2′,5′		
I-2′	116.9, CH	6.60, s	I-2,6′	I-2,6′	I-2,3,4, II-2
I-3′	145.3, C		I-2′,5′		
I-4′	145.5, C		I-2′,6′		
I-5′	117.6, CH	6.70, brd (8.0)		I-6′	I-6′
I-6′	122.7, CH	6.45, brd (8.0)	I-2,2′	I-2,2′,5′	I-2,3,4,5′, II-2
II-2	75.8, CH	4.88, s	II-3,4,2′,6′	II-3,4,2′,6′	I-2′,6′, II-3,2′,6′
II-3	70.8, CH	3.49, s	II-2,4	II-2,4	II-2,4,2′,6′, III-5′
II-4	37.8, CH	4.14, s	II-2,3	II-2,3	II-3
II-4a	105.0, C		II-3,4		
II-5	157.2, C		II-4		
II-6	98.6, CH	6.09, s			
II-7^d^	156.0, C				
II-8	110.6, C		I-3,4		
II-8a	152.9, C		I-4		
II-1′	132.5, C		II-2,3,5′		
II-2′	117.2, CH	6.75, s	II-2,6′	II-2,6′	II-2,3 III-2
II-3′	145.5, C		II-5′		
II-4′	145.2, C		II-2′,6′		
II-5′	117.6, CH	6.66, brd (8.0)		II-6′	II-6′
II-6′	121.3, CH	6.07, brd (8.0)	II-2,2′	II-2,2′,5′	II-2,3,5′, III-2
III-2	79.8, CH	4.81, s	III-4eq,2′,6′	III-3,4eq,2′,6′	I-2,2′, II-2′, III-3,2′,5′
III-3	66.0, CH	4.22, s	III-2,4eq	III-2,4eq,4ax	III-2,4eq,4ax,2′,6′
III-4eq	23.7, CH_2_	2.38, brd (16.8)	III-2	III-2,4ax	III-2,4ax (III-2′-neg)
III-4ax		1.95, brd (16.8)		III-3,4eq	III-2,4eq,2′
III-4a	101.1, C		III-3,4eq,4ax		
III-5	155.0, C		III-4eq		
III-6^d^	96.9, CH	5.93, s			
III-7	155.5, C		II-4		
III-8	110.1, C		II-3,4		
III-8a	152.9, C		III-2,4eq		
III-1′	132.9, C		III-2,5′		
III-2′	113.9, CH	5.91, s	III-2,6′	III-2,6′	III-2,3,4ax (III-4eq, neg)
III-3′	145.8, C		III-2′,5′		
III-4′	144.7, C		III-2′,6′		
III-5′	117.3, CH	6.27, brd (8.0)		III-2,6′	II-3, III-6′
III-6′	118.0, CH	5.09, brd (8.0)	III-2,2′	III-2,2′,5′	III-3,5′

a 500 MHz for ^1^H, 125 MHz for ^13^C.

b H (number) to C in the row.

c H (number) to H in the row.

d Assigned by comparison with compounds **2** and **3**.

To evaluate the contribution of each specific polyphenolic compound to the antidiabetic properties of the whole extract, the activity of these compounds was measured by a set of analyses. The capability of these compounds to activate insulin signaling was measured in Cho-IR cells, which are highly sensitive to the stimulation of insulin signaling. We first characterized the dose–response and time course of insulin signaling activation induced by insulin and SSE in these cells (Figure S4A,B, supplementary data). A dose-dependent effect of insulin-induced IR phosphorylation is presented, showing that 10 nM insulin is sufficient to achieve a maximal effect. IR phosphorylation was minimal with 1 nM insulin, although AKT phosphorylation was also highly stimulated upon this low-dose insulin treatment, suggesting that the limited extent of IR phosphorylation induced by 1 nM insulin is sufficient to induce maximal phosphorylation of AKT. SSE treatment stimulated IR phosphorylation only at the highest dose used (100 μg/mL), while AKT was phosphorylated in a dose-dependent manner within the 2–100 μg/mL range. IR phosphorylation was detected following insulin and SSE treatment at all time points measured (2–15 min, Figure S4B, supplementary data). Accordingly, all additional experiments were conducted following 10 min of incubation of insulin or SSE.

**Figure 4:**
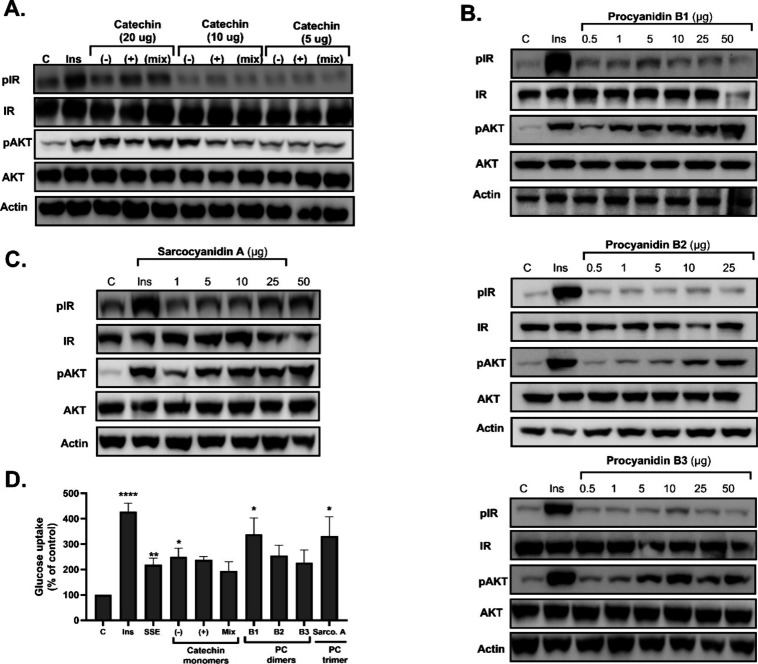
Bioactivity of monomer, dimer, and trimer catechins. (A) CHO-IR cells were treated with epicatechin [(−)-catechin], (+)-catechin, and a mix of these catechins (A), dimers of catechins (B), and with trimer catechin, sarcocyanidin A, isolated from SSE (C) for 10 min. Insulin (10 nM) was used as a positive control. Western blot analysis of the whole lysate was performed using specific antibodies. (D) Differentiated 3T3-L1 adipocytes were treated with SSE or with monomers, dimers, and sarcocyanidin A (**1**) at 100 μg/mL, with the use of insulin (100 nM) as a positive control. The uptake of [^3^*H*]-2-deoxy-d-glucose into cells was determined as described in Methods. The data represent the mean ± SEM of measurement made on at least three independent experiments. *p < 0.05, **p < 0.005, and ****p < 0.0001 compared to untreated cells by one-way ANOVA, followed by Tukey’s post-testing.

Monomeric, dimeric, and trimeric forms of PCs were detected in the active fraction of SSE. Among the monomers, (−)-epicatechin, isolated from the extract, was found to be effective in the stimulation of IR and AKT phosphorylation and glucose uptake, with higher potency than that of (+)-catechin (Figure 4A,D). Next, we compared the activity of PC-B1, which was detected in the extract, to other known dimers PC-B2 and PC-B3. The activity of PC-B1 was compared to other catechin dimers (PC-B2 and PC-B3); all were commercially purchased (Cayman Chemical, Ann Arbor, USA, purity >98%). PC-B1 was found to be the most effective among the three dicatechins investigated. This was concluded based on its capability to induce IR and AKT phosphorylation at lower doses than that of PC-B2 and B3 and to stimulate glucose uptake in 3T3-L1 adipocytes (Figure 4B,D). Lastly, the trimeric PC isolated, sarcocyanidin A (**1**), was highly effective in the induction of IR and AKT phosphorylation and stimulation of glucose transport (Figure 4C,D).

Previous studies demonstrated that the pentacyclic terpenoids tormentic acid and hydroxytormentic acid, ursolic acid, and hydroxy-ursolic acid are major components of SSE.^8^ Derivatives of tormentic acid were also detected by us by LCMS (Table 1 and Figure 2). Although these compounds were not detected in the active fractions (fractions 4 and 5), because of their high presence in SSE, as shown here for 23-HTAE, their bioactivity was also measured (Figure S5, supplementary data). Clearly, these molecules did not stimulate insulin signaling in CHO-IR cells. Therefore, their effect on glucose transport was not measured.

In the next part of the study, we investigated the mechanism of action of SSE and its active composite sarcocyanidin A (**1**). Our previous studies demonstrated that SSE activates insulin signaling through a PI3K-AKT-dependent pathway. In the next experiments, the assumption that IR is a direct target of SSE and is essential for SSE-dependent activation of insulin signaling was investigated.

**Figure 5:**
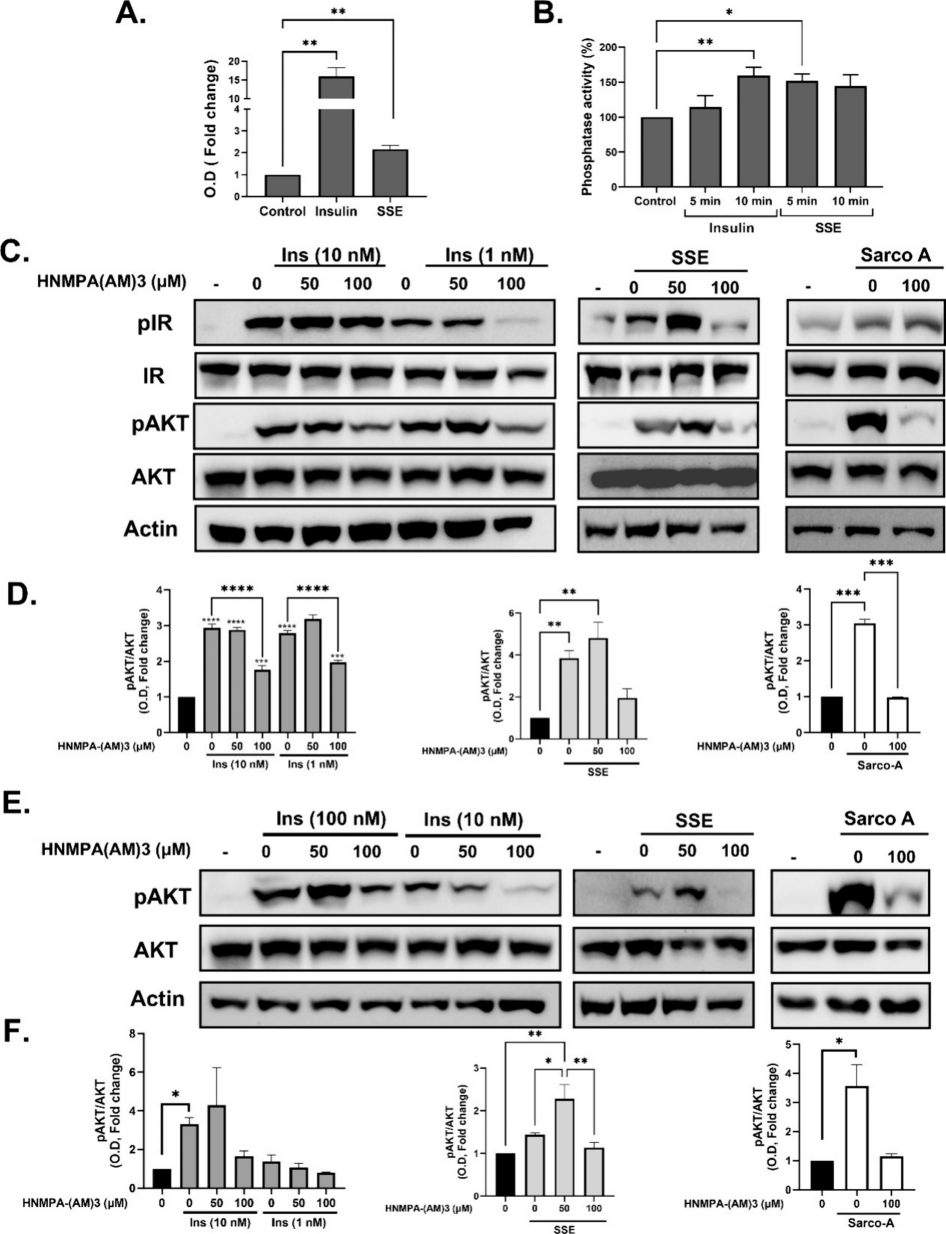
IR phosphorylation is required for SSE-induced AKT phosphorylation. (A) IR is phosphorylated on tyrosine residues by SSE. L6 myotubes were treated with 100 nM insulin or SSE (100 μg/mL) for 10 min. The cell lysate was prepared, and IR phosphorylation was measured by ELISA, according to the manufacturer’s instructions. (B) L6 myotubes were treated with 100 nM insulin or SSE (100 μg/mL) for the indicated time. Tyrosine phosphatase activity was measured as described in Methods. CHO-IR cells (C, D) and L6 myotubes (E, F) were treated with HNMPA-(AM)3 for 60 min, followed by treatment with insulin, SSE, or Sarco-A (100 μg/mL) for an additional 10 min. Western blot analysis of the whole lysate was performed using specific antibodies, and optical densitometry was measured by ImageJ. The data represent the mean ± SEM of measurement made on at least three independent experiments. *p < 0.05, **p < 0.005, compared to untreated cells or as indicated in the graphs by one-way ANOVA, followed by Tukey’s post-testing.

IR phosphorylation was measured by sandwich ELISA, in which the wells are coated with a specific anti-IR antibody, and phosphorylation of bound receptors is detected by an antibody specific for phosphorylated tyrosine. This approach is required to validate the specific phosphorylation of IR because of the cross-reactivity of the pIR antibodies with pIGF. As shown (Figure 5A), SSE induced a 2.17-fold increase in IR phosphorylation compared with a 15.89-fold increase by insulin.

The increase in IR phosphorylation might result from a direct binding and receptor activation or from inhibition of phosphorylated tyrosine phosphatase 1B (PTP1B), which is the specific phosphatase of IR. To elucidate whether the effect of SSE on IR is mediated through phosphatase inhibition, the activity of PTP was measured in L6 myocytes (Figure 5B). As observed, both insulin and SSE increased PTP activity, presumably representing the activation of the downregulated response required to attenuate insulin signaling following its stimulation. Therefore, it might be concluded that SSE-induced IR phosphorylation is not mediated through phosphatase inhibition and, hence, is presumably achieved through direct activation of the receptor by specific composites of SSE.

To clarify whether activation of IR mediates the stimulatory effect of SSE and trimeric sarcocyanidin A (**1**) on insulin signaling, phosphorylation of IR and AKT was measured in the presence of an IR inhibitor (HNMPA(AM)3) in both CHO-IR and L6 cells (Figure 5C-F). HNMPA(AM)3 at a concentration of 100 μM inhibited insulin-induced IR and AKT phosphorylation (1 or 10 nM insulin in CHO-IR or L6 cells, respectively). Interestingly, SSE-induced IR and AKT phosphorylation were stimulated with HNMPA(AM)3 given at a dose of 50 μM, while a higher dose (100 μM) abolished the SSE effects. Similarly, the phosphorylation of Akt, induced by sarcocyanidin A (**1**), was abolished by the IR inhibitor. These results indicate that insulin receptor activation mediates the SSE-induced AKT phosphorylation.

**Figure 6:**
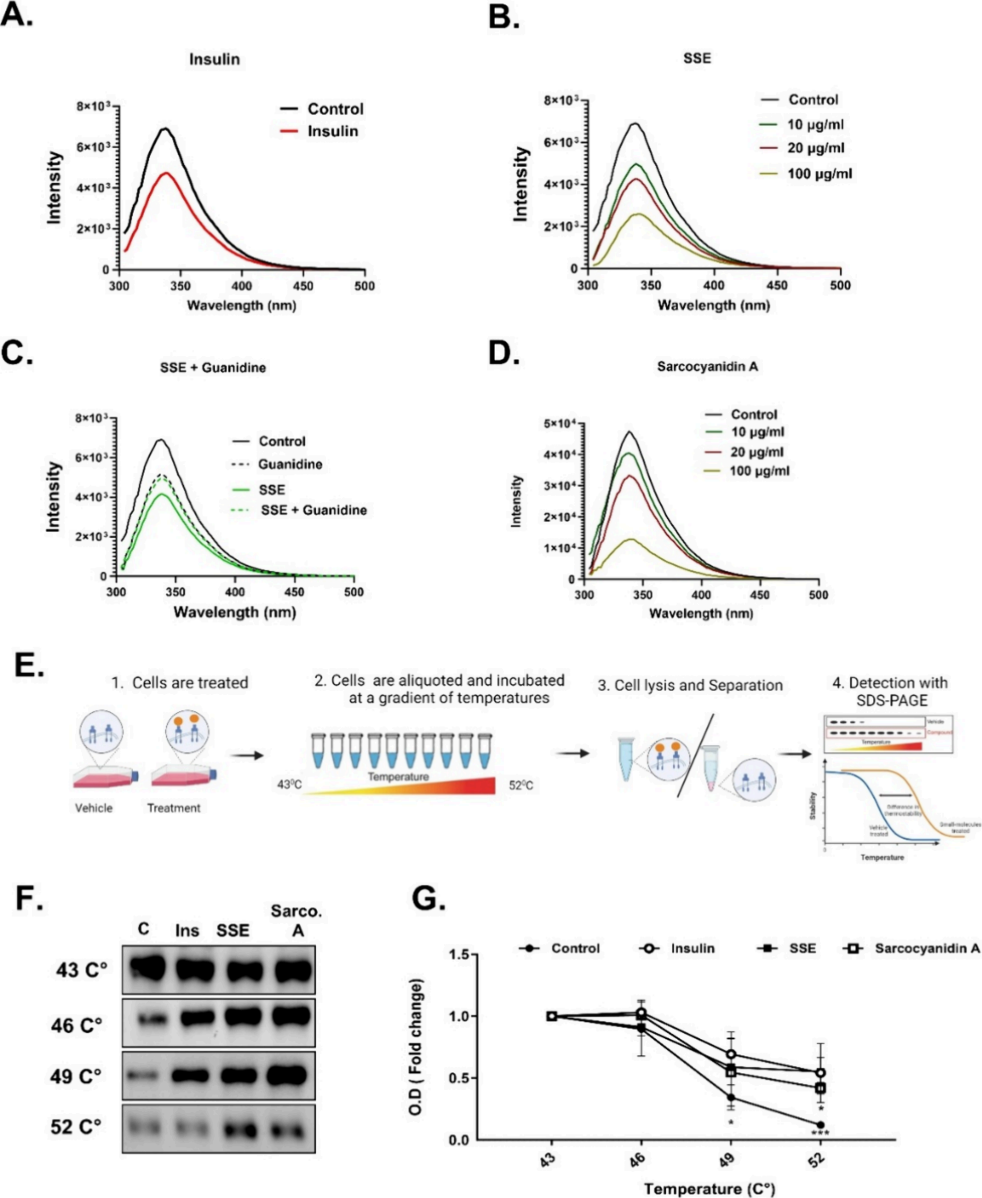
SSE directly interacts with IR. Autofluorescence of IR was measured in the presence of 100 nM insulin (A), SSE (B), SSE (20 μg/mL) in the presence of guanidine (1 M) (C), and sarcocyanidin A (**1**) (D). CHO-IR cells were treated with insulin (10 nM), SSE, or sarcocyanidin A (**1**, 10 μg/mL) for 10 min. A cellular thermal shift assay (CTSA) was conducted as described in Methods. (E) Schematic illustration of the protocol. (F) IR expression in lysates after incubation at a gradient of temp. This is a representative result of five independent experiments. Sarcocyanidin A (**1**): sarco-A. (G) Optical density of IR expression. *p < 0.05, ***p < 0.001, compared to OD of IR in untreated cells at the same temperature, by one-way ANOVA, followed by Tukey’s post-testing.

The presence of direct binding between SSE and IR was investigated by measurements of IR autofluorescence and by the cellular thermal shift assay (CTSA). Like the insulin effect, SSE attenuated IR autofluorescence in a dose-dependent manner, indicating that a composite of SSE directly binds IR, leading to a change in its conformation and the resulting autofluorescence (Figure 6A,B). This effect was abolished in the presence of the denaturizing agent guanidine, indicating that an accurate protein structure of IR is required for SSE binding (Figure 6C). A similar effect of a reduction in IR autofluorescence was observed with sarcocyanidin A (Figure 6D). The cellular thermal shift assay (CTSA) also validated the binding of IR to a certain composite of SSE. This assay is based on the principle of heat-induced protein denaturation, forming aggregates that sediment upon centrifugation, in contrast to native proteins that are soluble and preserved in the supernatant. The stability of proteins is expected to be enhanced upon binding to other molecules, leading to a delay in heat-dependent denaturation and sedimentation (Figure 6E).^18^ In this assay, CHO-IR cells were treated with SSE or sarcocyanidin A (**1**), and then, cell lysates were heated to different temperatures (43–52 °C). The denatured proteins were removed by centrifugation, and only the soluble proteins were analyzed by Western blotting. As presented in Figure 6F,G, heating the control lysates to temperatures over 43 °C led to a lower level of IR in the supernatant, indicating denaturation of the receptor at this temperature. However, pretreatment with either insulin, SSE, or sarcocyanidin A (**1**) increased the stability of IR to heat-induced denaturation, indicating the binding of sarcocyanidin A (**1**) or some additional components of SSE to the receptor.

**Figure 7:**
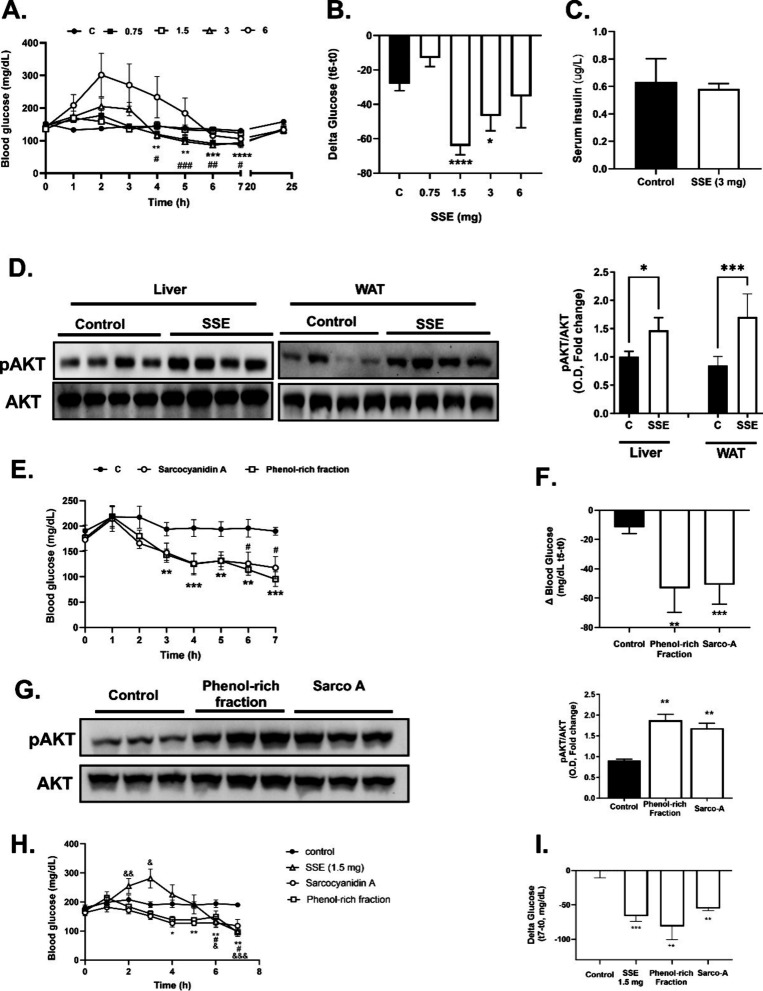
Effect of a single dose SSE, phenol-rich fraction, and sarcocyanidin A (Sarco-A) on blood glucose in mice. Male C57bl/6J were given an intraperitoneal administration of SSE. Blood glucose and serum insulin were measured once per hour for 7 h and an additional measure after 24 h (A). (B) The reduction in blood glucose occurred 6 h after SSE administration. (C) Plasma insulin 6 h after SSE administration. (D) Western blot analysis and densitometry of pAKT expression in the liver and subcutaneous white adipose tissue (SC WAT) of treated mice 6 h after SSE administration. In a different set of experiments, sarcocyanidin A or the phenol-rich fraction (0.4 μg) was administrated, blood glucose was measured (E, F), the liver was harvested, and Western blot analysis was performed (G). Male C57bl/6J were given HFD for 6 weeks before intraperitoneal administration of the SSE (1.5 mg/mL), Sarco-A, or phenol-rich fraction (0.4 μg each). Blood glucose was monitored for 7 h (H, I). The data represent the mean ± SEM of measurement made on at least three independent experiments. A, B, E, F, H, and I: *p < 0.05, **p < 0.005, ***p < 0.001, and ****p < 0.0005, compared to time 0 of the same group by paired *t*-tests. D and G were analyzed by one-way ANOVA, followed by Tukey’s post-testing.

The ability of SSE to activate insulin signaling was validated *in vivo.* SSE was administrated to normoglycemic C57bl/6 mice at increasing doses, and blood glucose was monitored (Figure 7A,B). A significant reduction in blood glucose was observed in SSE-treated mice after 5–7 h at doses of 1.5 and 3 mg, while it was not observed by 6 mg of SSE, a dose that even induced an elevation in blood glucose, presumably indicating a toxic effect of this dose. Glucose levels returned to a baseline after 24 h. An elevation in insulin levels did not accompany the reduction in blood glucose (Figure 7C), indicating that the glucose-lowering properties resulted from the activation of insulin signaling rather than stimulating the secretion of the hormone. This assumption is supported by the results of Western blot analysis (Figure 7D), showing a higher level of AKT phosphorylation in the liver and subcutaneous (SC) WAT in response to SSE administration. Similar results were obtained in response to administering the phenol-rich fraction and sarcocyanidin A (**1**) (Figure 7E-G). The glucose-lowering properties of SSE, the phenol-rich fraction, and sarcocyanidin A (**1**) were also demonstrated in HFD-fed mice (Figure 7H, I). While SSE induced an early hyperglycemic response, which precedes the glucose-lowering effect, this phenomenon was not found in response to phenol-rich fraction and sarcocyanidin A administration.

## Discussion

*Sarcopoterium spinosum* root extract is used by Bedouin traditional medicinal practitioners as an antidiabetic drug. Experiments conducted in cells and in mouse models support the potential therapeutic properties of this plant extract for the management of blood glucose.^11,12,14,15^ However, despite the progress achieved in the research of its antidiabetic properties, the active composites responsible for the therapeutic function of SSE have not been identified before. In this study, we successfully isolated the active fraction, identified a novel active compound, and demonstrated its direct stimulatory effect on the insulin receptor.

The bioguided fractionation approach revealed that the phenolic-rich fraction is responsible for the antidiabetic properties of SSE. This fraction stimulated the transmission of insulin signaling and induced glucose uptake in cells, even with a higher potency than achieved by the whole extract, indicating that components of this fraction are sufficient, while those of other fractions are not required, to achieve the insulin-mimetic properties of SSE.

Additional fractionation steps were conducted after identifying the phenol-rich fractions as the active ones. These fractions (combined fractions 4 + 5) were separated by size-exclusion chromatography to several subfractions, and their bioactivity was analyzed. This bioguided fractionation strategy enables the identification of active compounds, all of which are phenolics of the flavanol family. We found that monomers, dimers, and trimers of flavonols, including a novel procyanidin trimer, activated insulin signaling.

The major novelty of this study stems from the identification of a novel procyanidin trimer. This PC trimer, isolated from the SSE, was found to be highly effective in the induction of IR and Akt phosphorylation. This trimer was named sarcocyanidin A (**1**) and identified as (+)-catechin-4β-8-(−)-epicatechin-4β-8-(−)-epicatechin, which differs from the already known natural procyanidin trimers PC-C1 (an epicatechin trimer) and PC-C2 (a catechin trimer). In addition, it also differs from the procyanidin trimers epicatechin-(4β-8)-epicatechin-(4β-8)-catechin trimer and epicatechin-(4β-8)-catechin-(4α-8)-epicatechin trimer, which were synthetically attained with a condensation reaction.^20^ Therefore, the identification of sarcocyanidin A (**1**) in SSE is the first documentation of this PC trimer, either naturally achieved or synthetically achieved.

The monomer (−)-epicatechin was more effective than its enantiomer (+)-catechin, demonstrating a higher induction of Akt phosphorylation and glucose uptake. However, both of these two enantiomers did not stimulate the phosphorylation of IR over control. Among PC dimers, PC-B1, a dimer of (−)-epicatechin- and (+)-catechin, which was the PC detected in the active fraction of SSE, was the most effective PC dimer and the only dimer that successfully stimulated IR phosphorylation and glucose uptake.

These results, demonstrating that the dimer and oligomer of flavanols are more active in the activation of insulin signaling cascade than monomers, are in line with previous studies, showing that oligomeric PC-rich extracts stimulated glucose uptake through an IR and Akt-dependent mechanism.^21,22^ The oligomer-rich fraction isolated from cocoa beans (enriched for dimers and PCs with a 3–6 polymerization degree) was proven to be more effective in preventing glucose intolerance and insulin resistance in mice than either monomeric or polymeric-enriched fractions.^23^ These results support the advantage of dimeric and oligomeric PCs over monomers and polymers in activating insulin signaling.

However, while most studies investigated the potency of PC-rich fractions rather than isolated compounds,^21,22^ in our study, we could isolate and identify the specific PC dimer and trimer exerting the insulin-mimetic function. We found that PC-B1 is more potent than PC-B2 and PC-B3 in activating insulin signaling. To the best of our knowledge, there has been no previous study in which the insulin-mimetic activity of PC dimers was compared. The identification of PC-B1 in SSE, rather than the less potent PC-B2 and PC-B3, might explain its high efficacy as an antidiabetic plant. Regarding the trimer, a previous study demonstrated that PC-C1, an epicatechin trimer, induced Akt phosphorylation and glucose transport and facilitated glucose disposal from the blood after glucose load more efficiently than the monomer and dimer of epicatechin.^24,25^ However, the effect of PC-C1 on IR phosphorylation was not presented, in contrast to our data, demonstrating that the SSE-isolated trimer (+)-catechin-4β-8-(−)-epicatechin-4β-8-(−)-epicatechin mimics insulin action through its binding to the IR.

Interestingly, the pentacyclic triterpenes tormentic and ursolic acids, known constituents of SSE,^8^ did not activate insulin signaling at all. Several biological activities had been attributed to tormentic and ursolic acid in previous studies, including hypoglycemic properties, which are achieved through stimulation of insulin secretion.^26−29^ The capability of SSE to induce insulin secretion in a cell line of beta cells was demonstrated by us in a previous study.^12^ However, *in vivo* experiments conducted on mouse models of glucose intolerance and type 2 diabetes clearly demonstrated that the antidiabetic properties of SSE are mainly achieved through amelioration of insulin sensitivity rather than stimulating insulin secretion.^11,13,14^ We assume that the positive effects of tormentic and ursolic acid on managing blood glucose in insulin-resistant conditions stem from the anti-inflammatory properties of these compounds.^27,30−34^ This hypothesis should be tested in future studies.

Since T2D is accompanied by an elevated, chronic inflammation, which worsens the metabolic alterations and promotes the development of diabetic complications, molecules with anti-inflammatory properties might work synergistically with insulin-mimetic molecules to improve the metabolic health of people with T2D.^35^ The unique composition of SSE, including insulin-mimetic compounds and anti-inflammatory characteristics, as demonstrated in our previous studies,^14,15,36^ provides this extract its high antidiabetic potency. However, SSE likely contains additional components that either do not contribute to its glucose-lowering effects or may even counteract the actions of sarcocyanidin A, PC-B1, and other antidiabetic compounds within the extract. Notably, administration of the isolated active fraction or sarcocyanidin A (**1**) to mice enhances the glucose-lowering effect of *Sarcopoterium spinosum*. Therefore, identifying the active fraction and active molecules, achieved in this study, may facilitate the development of more potent SSE-based antidiabetic formulations.

In this study, we demonstrated that IR is a target of SSE and sarcocyanidin A (**1**). IR inhibition abrogated SSE-induced phosphorylation of Akt, demonstrating the role of IR in mediating the SSE effect on insulin signaling. A direct binding of SSE and sarcocyanidin A (**1**) was demonstrated by CTSA, in which an elevated thermal stability of IR was achieved in the presence of SSE and the trimeric PC. In addition, autofluorescence of native IR was reduced in the presence of SSE and the trimeric PC, while this was not observed with denaturized IR, indicating that the three-dimensional structure of IR is necessary to enable binding. This result of IR being activated by sarcocyanidin A (**1**) is in line with a previous study, showing a similar effect of grapeseed procyanidin extract (GSPE)^21^. However, while the GSPE is a mix composed of monomers, dimers, trimers, tetramers, and longer oligomers, our data demonstrated for the first time the stimulatory effect of an isolated procyanidin trimer on IR phosphorylation.

In summary, this study identified the active components of the SSE. PC-B1 and the novel PC trimer sarcocyanidin A (**1**) are the most potent of these molecules, leading to the activation of insulin signaling. The insulin receptor is stimulated by sarcocyanidin A (**1**). Binding properties should be investigated in future studies to identify the binding site and its affinity.

## Supporting Information Available

(Scheme S1) Scheme of the separation of compounds **1**–**4** from the crude SSE; (Figure S2) structure assignment of sarcocyanidin A (**1**), supported by the HMBC correlations; (Figure S3) structure assignment of sarcocyanidin A (**1**), supported by the NOEs from a ROESY experiment; (Figure S4) dose–response and time course of SSE-induced IR and AKT phosphorylation; (Figure S5) tormentic and ursolic acids that did not stimulate insulin signaling; (Table S1) 2D NMR data of (−)-epicatechin (**2**) in DMSO-*d*_6_; (Table S2) 2D NMR data of (+)-catechin (**3**) in DMSO-*d*_6_; (Table S3) procyanidin B1 (**4**) in acetone-*d*_6_ (PDF)

## Abbreviations Used

CHO-IR: CHO cells overexpressing the insulin receptor
CTSA: cellular thermal shift assay
DTT: dithiothreitol
HFD: high fat diet
HNMPA(AM)3: hydroxy-2-naphthalenylmethylphosphonic acid triacetoxymethyl ester
IR: insulin receptor
PC: procyanidin
PTP: phospho-tyrosine phosphatase
RIPA: radioimmunoprecipitation assay
SC WAT, SDS: sodium dodecyl sulfate
SSE: sarcopoterium spinosum extract
T2D: type 2 diabetes
