# Supplementary material for: Activation of the Insulin Receptor by Sarcopoterium spinosum Extract and Identification of Sarcocyanidin A as a Novel Active Compound
Source: ACS Omega. 2025 Apr 9;10(15):15511–24. doi: 10.1021/acsomega.5c00451 (PMC12019437; doi:10.1021/acsomega.5c00451)
Supplement: Supplementary file 1 — ao5c00451_si_001.pdf [file ao5c00451_si_001.pdf]

Activation of insulin receptor by Sarcopoterium spinosum extract and identification of Sarcocyanidin A as a novel active compound

Ayala Wollman[a#], Rania Hasib Afana[b#], Shmuel Carmeli[b] and Tovit Rosenzweig[a,c*]

[a] Department of Molecular Biology, Faculty of Natural Sciences, Ariel University, Israel.

[b] Raymond and Beverly Sackler School of Chemistry, Faculty of Exact Sciences, Tel Aviv University, Ramat Aviv, Israel.

[c] The Adelson School of Medicine, Ariel University, Israel.

[#]Equal contribution

[*]Corresponding author

Supplementary data:

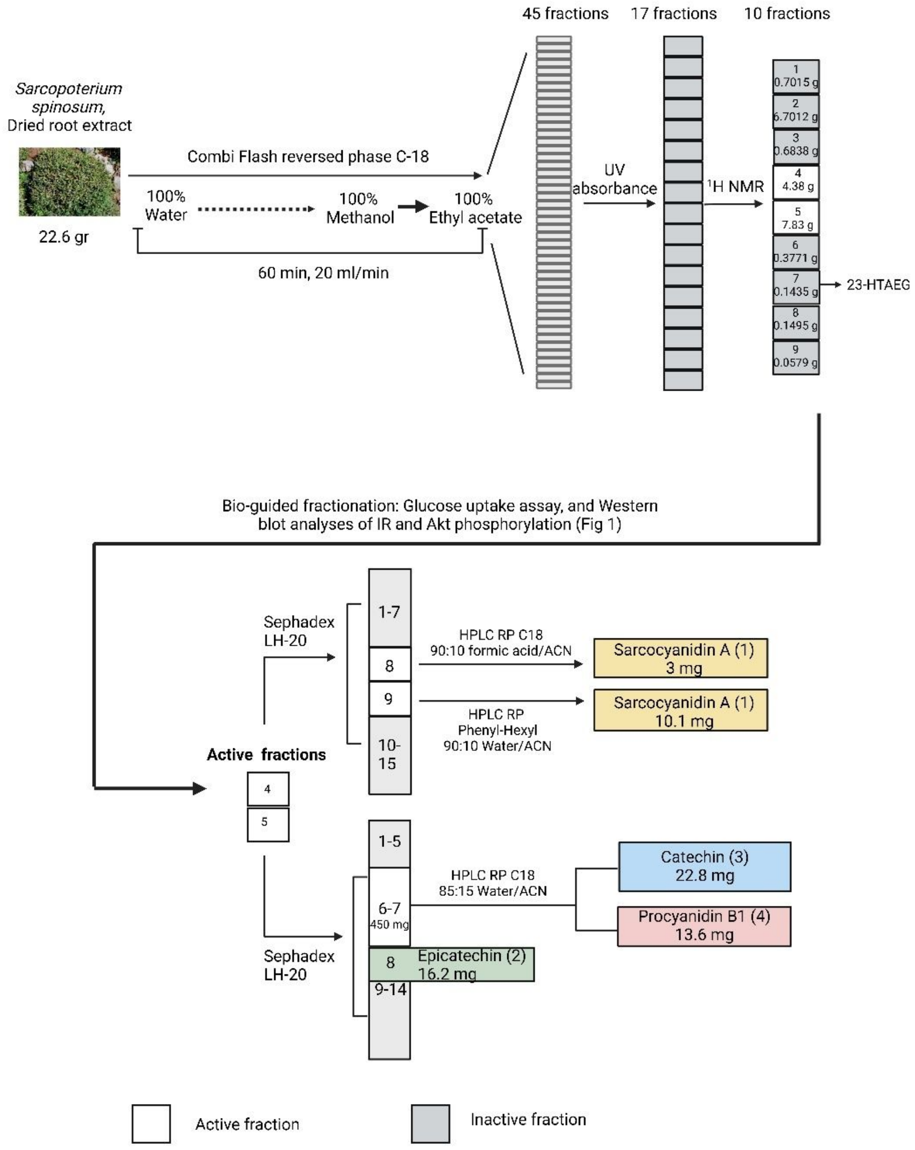

Scheme S1. A scheme of the separation of compounds **1-4** from the crude SSE.

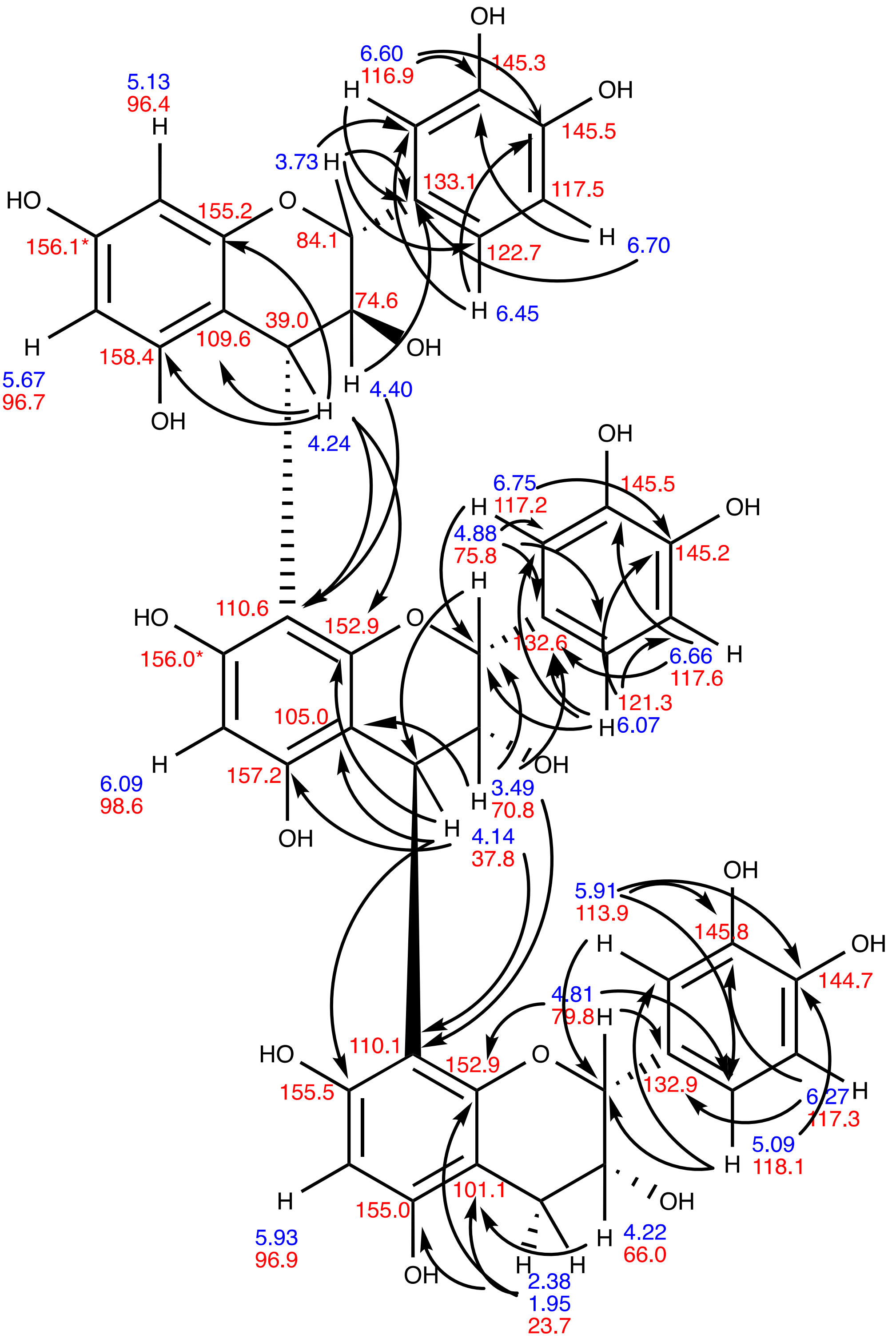

Figure S2. Structure assignment of Sarcocyanidin A (**1**), supported by the HMBC correlations.

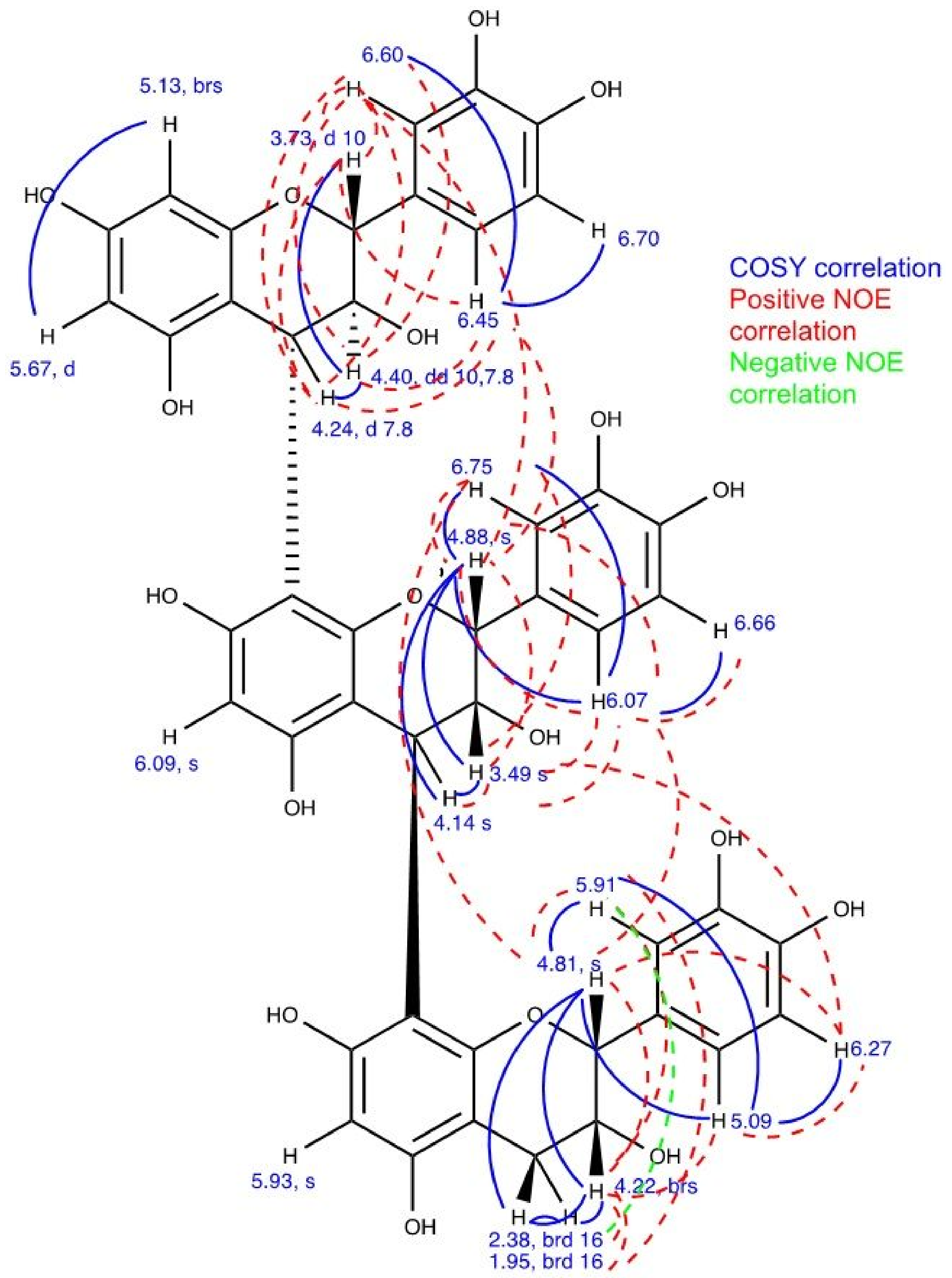

Figure S3. Structure assignment of Sarcocyanidin A (**1**), supported by the NOE's from a ROESY experiment.

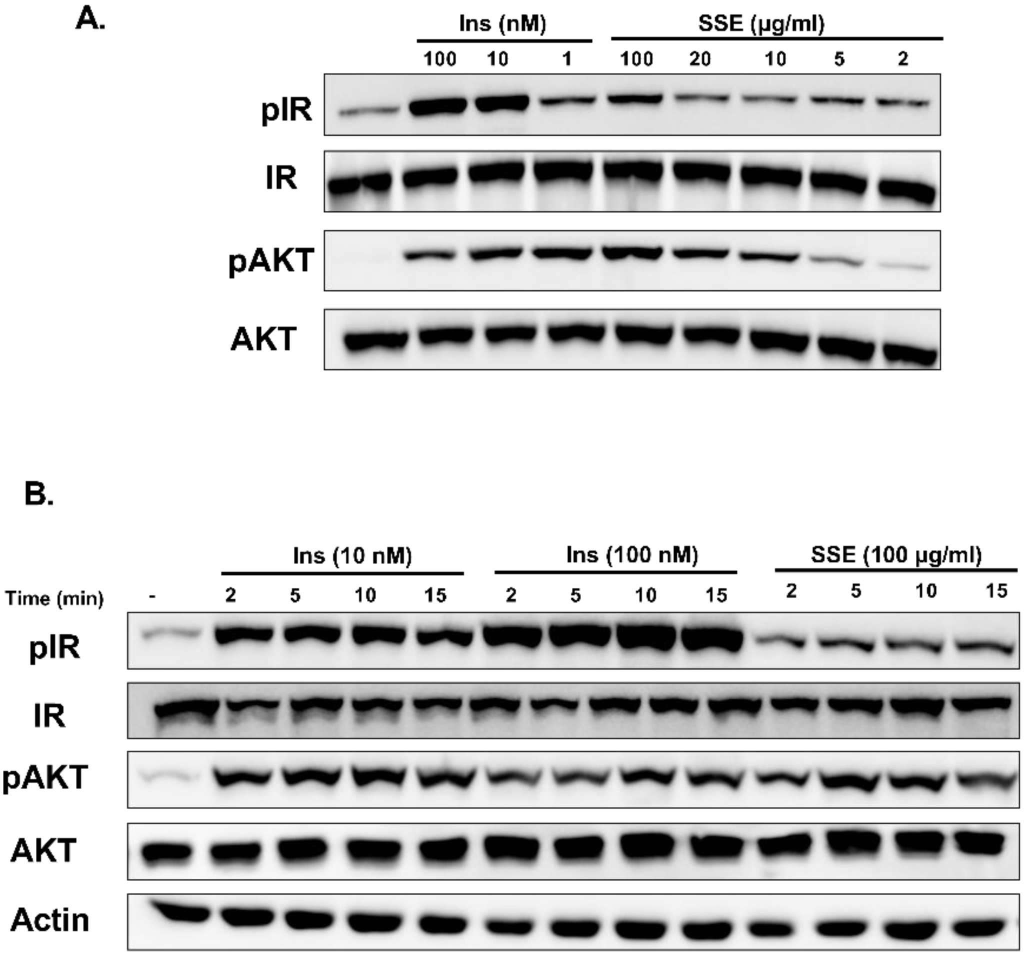

Figure S4. Dose-response and time course of SSE-induced IR and AKT phosphorylation. A. CHO-IR cells were treated with insulin or SSE at the indicated doses for 10 min. B. CHO-IR cells were treated with insulin (10 and 100 nM) or SSE (100µg/ml) for the indicated time. A&B. Western-blot analysis of whole lysate was performed using specific antibodies.

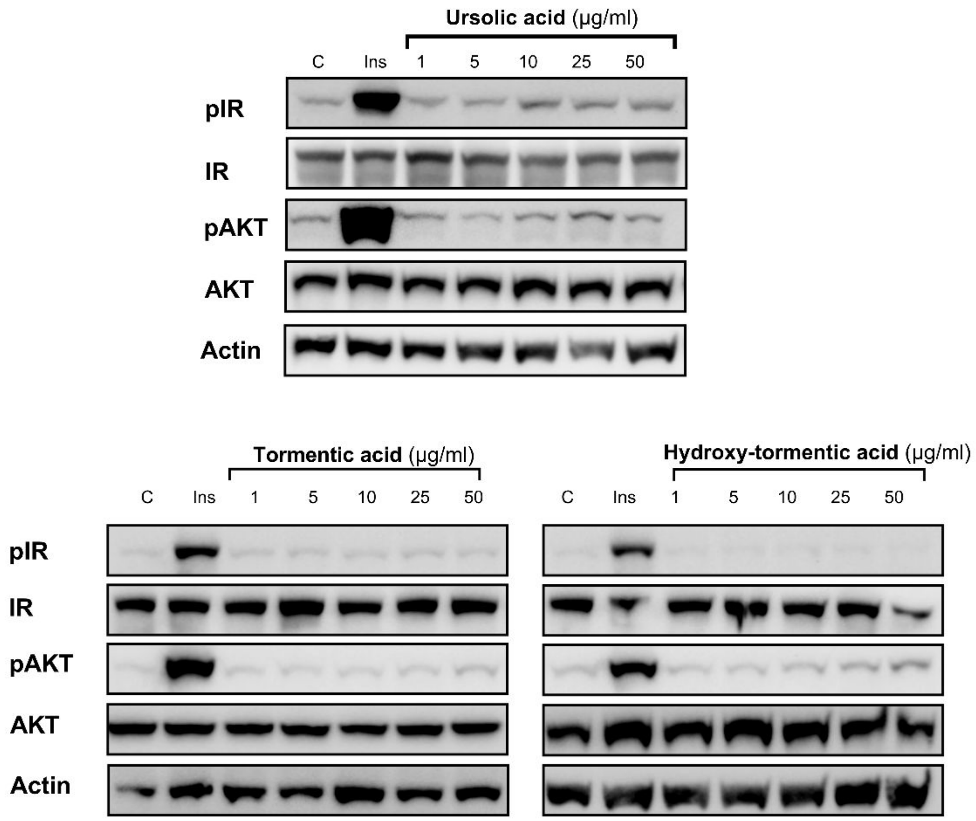

Figure S5. Tormentic and Ursolic acids did not stimulate insulin signaling. A. CHO-IR cells were treated with ursolic, tormentic or hydroxy-tormentic acids at the indicated doses for 10 min. Insulin (10 nM) was used as a positive control. Western-blot analysis of whole lysate was performed using specific antibodies.

Table S1. 2D NMR Data of (-)-epicatechin (**2**) in DMSO-d_6[a]

Position	δ_C, mult.	δ_H, mult. (J in Hz)	HMBC correlations[b]	COSY correlations[c]	TOCSY correlations[c]	ROESY correlations[c]
2	78.3, CH	4.72, brs	4a,4b,2’,6’	3,4a,4b	3,4a, 4b	3,4a,2’,6’
3	65.2, CH	3.99, dd (4.4, 3.0)	4a,4b	2,4a,4b	2,4a, 4b	2,4a,4b,2’,6’
4a	28.5, CH_2	2.66, dd (16.5, 4.4)	2,3	2,3,4b	2,3,4 b	2,3,4b
b		2.47, dd (16.6, 3.0)		2,3,4a	2,3,4 a	3,4a
4a	98.8, C		3,4a,4b,6,8			
5	156.8, C		4a,4b,6,8			
6	95.3, CH	5.88, d (1.8)	4a,4b,8	8	8	
7	156.5, C		6			
8	94.3, CH	5.71, d (1.8)	4a,6	6	6	
8a	156.0, C		2,4a,4b,8			
1’	130.9, C		2,3,4b,2’,5’,6’			
2’	115.1, CH	6.88, brs	2,6’	6’	6’	2,3
3’	144.7, C		2’,5’			
4’	144.8, C		2’,5’			
5’	115.0, CH	6.66, d (8.2)	2’	2,6’	2,6’	
6’	118.2, CH	6.64, dd (8.2, 1.1)	2,2’	2,2’,5’	2,2’, 5’	2,3

[a]500 MHz for ^{1}H, 125 MHz for ^{13}C; [b]H (number) to C in the row; [c]H (number) to H in the row.

Table S2. 2D NMR Data of (+)-catechin (**3**) in DMSO-d_6[a]

Position	δ_C, mult.	δ_H, mult. (J in Hz)	HMBC correlations[b]	COSY correlations[c]	TOCSY correlations[c]	ROESY correlations[c]
2	81.2, CH	4.46, d (7.0)	4eq,4ax,2’,6’	3,2’,6’	3,3-OH,4eq, 4ax,2’,6’	4eq,2’,6’
3	66.5, CH	3.80, dddd (8.1,7.0,5.1, 3.8)	2 ,4eq,4ax	2,3-OH, 4eq,4ax	2,3-OH, 4eq,4ax	3-OH,4eq, 4ax, 2’,6’
3-OH		4.85, brd (3.8)		3	2,3,4eq,4ax	3
4eq	28.1, CH_2	2.64, dd (15.9, 5.1)	2	3,4ax	2,3,3-OH, 4ax	2,3
ax		2.33, dd (15.9, 8.1)		3,4eq	2,3,3-OH, 4eq	3,
4a	99.3, C		4eq,4ax,6,8			
5	156.7, C		4eq,4ax,6			
6	95.3, CH	5.87, d (2.0)	8	8	8	5-OH,7-OH
7	156.4, C		6,8			
8	94.0, CH	5.67, d (2.0)	6	6	6	7-OH
8a	155.6, C		2,4eq,4ax,6,8			
1’	130.8, C		2,5’			
2’	114.8, CH	6.71, d (1.7)	2,5’,6’	2,6’	2,6’	
3’	145.0, C		5’			
4’	145.0, C		2’,6’			
5’	115.3, CH	6.67, d (8.1)		6’	6’	6’
6’	118.6, CH	6.58, dd (8.1, 1.7)	2,2’	2,2’,5’	2,2’,5’	5’
3’,4’-OHs		8.85, brs				
7-OH		8.94, brs				6,8
5-OH		9.19, brs				6

[a]500 MHz for ^{1}H, 125 MHz for ^{13}C; [b]H (number) to C in the row; [c]H (number) to H in the row.

Table S3. Procyanidin B1 (**4**) in Acetone-d_6[a]

Position	δ_C, mult.	δ_H, mult. (J in Hz)	HMBC correlations[b]	COSY correlations[c]	COSY correlations[c]
I-2	77.1, CH	5.09, brs	I-3,4,2',5',	I-3,2'	I-3,2',5',
I-3	72.7, CH	3.97, brs	I-4	I-2,3-OH,4	I-2,4,2',5'
I-3-OH		3.50 brs			
I-4	37.0, CH	4.69, brs	I-3	I-2,3	I-3,3-OH
I-4a	101.1, C		I-3,4		
I-5	156.1, C		I-4		
I-6[d]	95.9, CH	5.95, s			
I-7	158.6, C				
I-8[d]	97.1, CH	5.95, s			
I-8a	157.9, C		I-4		
I-1'	132.6		I-2,2',5',		
I-2'	115.4, CH	6.99, brs	I-2,5'		I-2,3
I-3'	145.6, C		I-2',5'		
I-4'	145.5, C		I-2'		
I-5'	115.8, CH	6.73, m	I-2,2'		
I-6'	119.5, CH	6.82, m	I-2'	I-5'	I-3
II-2	82.3, CH	4.78, brm	II-4eq,ax		
II-3	68.0, CH	4.06, brm	II-4eq,ax	II-4eq,ax	II-2'
II-4eq	27.9, CH_2	2.78, dd (17.0, 6.7)		II-3,4ax	II-3,4ax
4ax		2.60, dd (17.0, 4.5)		I-3,4eq	II-3,4eq
II-4a	101.1, C		II-4eq,ax		
II-5	155.5, C		II-4eq,ax		
II-6[d]	96.4, CH	6.02, s			
II-7	155.5, C				
II-8	107.6, C		I-4		
II-8a	154.2, C		I-4,4eq,ax		
II-1'	132.4, C		II-5'		
II-2'	114.8, CH	6.91, brs	II-5'		II-3
II-3'	145.5, C		II-5',6'		
II-4'	145.4, C		II-2',6', II-5		
II-5'	115.6, CH	6.74, m	II-6'		
II-6'	119.4, CH	6.73, m	II-2'		

[a]500 MHz for ^{1}H, 125 MHz for ^{13}C; [b]H (number) to C in the row; [c]H (number) to H in the row. [d]assigned by comparison with compounds **2** and **3**.
